# Handbuch Der Praktischen Chirurgie Für Artze Und Wundärtze

**Published:** 1858-09

**Authors:** 


ART. VIII.—*Handbuch der Praktischen Chirurgie für Artze und Wundärzte.* Manual of Practical Surgery. By VICTOR VON BRUNS, M.D., Professor of Surgery in the University of Tübingen. Sections first and second; eight numbers. pp. 1640, 8vo. Tübingen, 1853–4–7.

THE King of Wurtemberg, in whose dominions the University of Tübingen is situated, an institution which has been the pride of his State and the glory of Germany for the last half century, was the first to transplant some of the disciples of the Vienna School to his alma mater, the fame of which he has ever been zealous to preserve and extend. He charged his commissioners to invite four of the most talented pupils of Rokitansky to take chairs in the university. The selection consisted of Bruns, Roser, Oesterlein, and Wunderlich, now a bright galaxy, stars of the first magnitude in the Esculapian constellation. Our author was found engaged in the preparation of a work on general anatomy, which, though the product of a mere medical student, a lad in appearance, created a profound sensation; abounding, as it did, in microscopical discoveries and new facts on analytical organic chemistry. Dr. Bruns was installed as professor in ordinary of surgery in 1852, and, with his colleagues, just arrived from Vienna, forthwith promulgated its principles. The title-page of the work informs us, by the appended "von" to the author's name, that he has lately been ennobled—an act of gracefulness which does more honor to the king who conferred it than to the recipient, whose fame certainly rests on a more imperishable basis. The Manual of Surgery bids fair to be a copious one; the four volumes now published, containing upwards of seventeen hundred octavo pages, treat only of the special surgery of the brain and its coverings, along with the organs of mastication and taste.

The English reader, knowing the proneness of the Germans to be too speculative and prolific in their writings, would naturally infer an unnecessary and confounding prolixity at the sight of these large volumes, comprising matter which he is wont to see dispatched with perhaps one-eighth the expenditure of paper. A short perusal, however, of their pages soon dispels these apprehensions, as the style is essentially English in clearness, conciseness, and terseness. The work is designed to be strictly practical; and this aim is never lost sight of, every line embodying facts as observed by himself or others, or rigidly inferred by an "*a posteriori*" mode of reasoning. Whatever is hypothetical is but cursorily noticed as an *on dit.*

The scheme itself on which the book is written tends to facilitate the study of its subjects, rendering it a complete repertory and codex, wanting only an alphabetical arrangement to make it an Encyclopædia of Surgery. The description of diseases is always preceded by their pathological anatomy, as observed, for the most part, by the author himself; and great stress is laid on this as the brilliant side of science. Etiology, Symptomatology, and Therapeutics follow. Each particular case is accompanied by illustrative observations, gleaned from his own experience, or from that of reliable authorities of every nation. Special attention is also given to medico-legal considerations wherever they can be fitly introduced.

The work comprises, first, General Surgery; under which head are described the materials, bandages, instruments, and apparatuses, as well as the employment of surgical operations, with rules and precepts to be observed in their performance. Secondly, General Surgical Pathology and Therapeutics, and the description of the processes which induce structural changes, aside from local modifications. Thirdly, the surgical affections of each separate region of the body, preceded by an anatomical description of each region.

The following singular case of osteatome of the scalp is the only one on record, and we therefore deem it unnecessary to apologize for presenting it to our readers. The subject, a man eighty years of age, was admitted into the Obuchoff Hospital, St. Petersburg, laboring under a tumor situated on the occiput, which he had had since his sixth year. He stated that in his infancy he was affected with tinea capitis, that suddenly disappeared, but was soon followed by a tumor, which, being treated by warm fomentations, softened, broke, and suppurated for a long time, but finally closed, its volume being at the time somewhat diminished. When he was twenty years old a metallic ligature was cast around its base, but violent inflammation supervening, further persistence was deemed impracticable. The tumor was then still movable, but soon became adherent to the cranium. He refused having it interfered with, and bore the inconvenience with patience until his death, which ensued soon after his admittance into the hospital, in consequence of cerebritis. The tumor measured, in its greatest circumference, two feet; from one ear to the other, twenty inches; at its base, fifteen and a half inches; and from apex to base, one foot seven inches; its weight was ten pounds. It received its covering from the widely-distended scalp, upon which the hair stood in isolated patches. The skin formed folds near the nape of the neck, with grooves three inches deep. The inner substance of this mass consisted of an ivory formation, with cells containing a yellow, spermaceti-like matter. The aponeurosis of the head formed folds on the base of the tumor, from which innumerable vessels penetrated the bony mass. The occipital bone had been entirely absorbed from the pressure of the tumor, and the first two vertebræ were reduced to a bony plate.

The diagnosis and treatment of fractures of the skull, as well as their bearing on forensic medicine, are fully and ably considered. The statistics on the subject of hare-lip are interesting; and we regret that want of space compels us to close this brief notice of a ponderous but highly valuable work, which should find a place in the library of every surgeon.

